# Adaptive wavelet‐VNet for single‐sample test time adaptation in medical image segmentation

**DOI:** 10.1002/mp.17423

**Published:** 2024-10-01

**Authors:** Xiaoxue Qian, Weiguo Lu, You Zhang

**Affiliations:** ^1^ Department of Radiation Oncology University of Texas Southwestern Medical Center Dallas Texas USA

**Keywords:** medical image segmentation, test‐time adaptation (TTA), unsupervised learning, wavelet transform

## Abstract

**Background:**

In medical image segmentation, a domain gap often exists between training and testing datasets due to different scanners or imaging protocols, which leads to performance degradation in deep learning‐based segmentation models. Given the high cost of manual labeling and the need for privacy protection, it is often challenging to annotate the testing (target) domain data for model fine‐tuning or to collect data from different domains to train domain generalization models. Therefore, using only unlabeled target domain data for test‐time adaptation (TTA) presents a more practical but challenging solution.

**Purpose:**

To improve the segmentation accuracy of deep learning‐based models on unseen datasets, and especially to enhance the efficiency and stability of TTA for individual samples from heterogeneous domains.

**Methods:**

In this study, we proposed to dynamically adapt a wavelet‐VNet (WaVNet) to unseen target domains with a hybrid objective function, based on each unlabeled test sample during the test time. We embedded multiscale wavelet coefficients into a V‐Net encoder and adaptively adjusted the spatial and spectral features according to the input, and the model parameters were optimized by three loss functions. We integrated a shape‐aware loss to focus on the foreground segmentations, a Refine loss to correct the incomplete and noisy segmentations caused by domain shifts, and an entropy loss to promote the global consistency of the segmentations. We evaluated the proposed method on multidomain liver and prostate segmentation datasets to assess its advantages over other TTA methods. For the source domain model training of the liver dataset, we used 15 3D MR image samples for training and 5 for validation. Correspondingly, for the prostate dataset, we used 22 3D MR image samples for training and 7 for validation. In the target domain, we used a single 3D MR image sample for adaptation and testing. The total number of testing samples is 60 in the liver dataset (for 3 different domains) and 116 in the prostate dataset (for 6 different domains).

**Results:**

The proposed method showed the highest segmentation accuracy among all methods, achieving a mean (± SD) Dice coefficient (DSC) of 78.10 ± 5.23% and a mean 95th Hausdorff distance (HD95) of 15.52 ± 5.84 mm on the liver dataset; and a mean DSC of 80.02 ± 3.89% and a mean HD95 of 9.18 ± 3.47 mm on the prostate dataset. The DSC is 11.67% (in absolute terms) and 15.27% higher than that of the baseline (no adaptation) method, for the liver and the prostate datasets, respectively.

**Conclusions:**

The proposed adaptive WaVNet enhanced the image segmentation accuracy from unseen domains during the test time via unsupervised learning and multi‐objective optimization. It can benefit clinical applications where data are scarce or with changing data distributions, including online adaptive radiotherapy. The code will be released at: https://github.com/sanny1226/WaVNet.

## 1 INTRODUCTION

Deep neural networks have significantly improved the accuracy of medical image segmentation,^1^, ^2^, ^3^, ^4^ while their performance heavily relies on the independent and identically distributed (i.i.d.) assumption of the training and testing data.^5^, ^6^, ^7^, ^8^ In clinical practice, the acquisition of medical images often involves different scanner hardware, imaging protocols, and patient populations, which inevitably introduce domain shifts. When a model trained on a source‐domain dataset was tested on another dataset from unseen target domains, its performance typically declines due to the domain gap and distribution variations.

To tackle this problem, different transfer learning, domain generalization, and domain adaptation methods have been proposed in recent years.^9^, ^10^, ^11^, ^12^ Transfer learning is the most straightforward approach by fine‐tuning a source model using labeled data from a new target domain.^13^, ^14^, ^15^ For instance, Karimi et al. fine‐tuned a source segmentation model on a new target‐domain task, by freezing the filters of the encoder of their network and reusing the features in deeper layers.^16^ However, transfer learning can be restricted by the availability of high‐quality, labeled target‐domain data, especially under scenarios with diverse, and changing clinical hardware/protocols.

Domain generalization typically aims to train a model on multiple source domains to achieve generalization to target domains. Various methods, including data augmentation,^17^ domain alignment,^18^ meta‐learning,^19^ and self‐supervised learning,^20^ have been employed to facilitate domain generalization. Zhang et al. proposed a deep stacked transformation approach, trying to address the domain shifts by data augmentation to simulate training data from multisource domains to derive a domain‐robust model.^21^ However, prior knowledge of potential domain shifts and distributions is essential for the domain generalization approaches, and the generalization ability can still be suboptimal for a specific target domain with unseen characteristics. It can also be challenging to curate high‐quality data from multiple source domains to train such generalized models.

Similarly, domain adaptation usually necessitates simultaneous access to both the source and target domains, which can be hard to achieve due to privacy concerns. Jiang et al. proposed a self‐derived organ attention deep learning network for magnetic resonance imaging (MRI) segmentation, where they achieved computed tomography (CT)‐to‐MRI domain adaptation by simultaneously training a CT‐to‐MR translation model and an MRI segmentation model using CT segmentation labels.^22^ This approach needs images from both the source (CT) and target (MRI) domains for image‐to‐image translation. Another study proposed a multisource domain adaptation strategy for segmentation and designed a multimodel consistency loss to transfer the learned multisource knowledge to the target domain.^23^ In clinical practices, labeling costs, data scarcity, and data privacy concerns often render the above‐mentioned methods impractical.

Different from the aforementioned methods, test‐time adaptation (TTA) does not require pretraining annotations of the target domains.^24^, ^25^, ^26^, ^27^, ^28^, ^29^, ^30^ Instead, it automatically optimizes the pretrained source domain model for unlabeled test domain samples, without accessing the source domain data during adaptation. Due to these characteristics, TTA makes a highly convenient and attractive approach to address domain shift problems. Due to the absence of labels at test time, different unsupervised losses have been proposed to guide the TTA model optimization. Wang et al. proposed a TTA method by introducing a loss function based on entropy minimization (TENT).^30^ The study verified that the entropy loss is positively correlated with the classification error, thus they updated the batch normalization layers of the model during the test time, by minimizing the entropy of model predictions on the target data to improve the segmentation results. However, test‐time entropy minimization (TENT) needs to use the whole test dataset rather than a single test time sample for adaptation, while the full dataset may not be available in clinical practices during the test time. TENT also exhibits instability when the target data have mixed distribution shifts.^31^ To address this limitation, several methods have been proposed to capture higher‐order information from data to achieve more stable performance. Bateson et al. proposed a weighted Shannon entropy and a class‐ratio loss for source‐free domain adaptation.^24^ Similarly, Niu et al. introduced a reliable sharpness‐aware entropy loss to improve the performance of TENT.^31^ Hu et al. designed a regional nuclear‐norm (RN) loss and a contour regularization (CR) loss to update the parameters of a pretrained source model during the TTA.^27^ The RN loss minimizes the nuclear norm on the segmentation classification matrix to promote a low‐rank representation to better distinguish different segmentation classes, while the CR loss measures the relevance between the pixel and its neighbors to enhance the segmentations’ continuity and connectivity. However, similar to the original TENT study, most of these methods require the whole test‐time dataset for adaptation, while adaptations using a single unlabeled test sample are more convenient and clinically feasible. Zhang et al. proposed a single‐sample TTA method by addressing unexpected domain variations through performing data augmentations on the input and adapting the model parameters by minimizing the entropy of the model's average, or marginal, output distribution across these augmentations.^32^ It reduced the domain variance by minimizing entropy to achieve consistent predictions across different augmentations of a single sample. This method is similarly based on the entropy minimization, and suffers from the same instability issues as TENT. In addition, its efficacy is further limited by the single‐sample‐based adaptation, as the single‐sample‐driven augmentation may not reflect the same data diversity as seen by the population‐based TENT method. In addition to designing various loss functions, some methods optimize the pretrained segmentation model by incorporating additional modules. The DAE‐based method^28^ trained a separate denoising autoencoder (DAE) to adapt an image normalization module to achieve single‐sample TTA. The DAE enables the segmentation network to produce “corrected” segmentation results. Although this approach enhanced performance in the target domain by denoising segmentation results, the efficacy is limited by the selected noise for training the DAE. Guo et al. proposed an atlas‐guided, single‐sample TTA method,^26^ in which they trained a registration network and an atlas map in the source domain, and utilized the atlas to provide supervision when adapting attention blocks in the segmentation network during the test time. Yet, the segmentation results can be easily overfitted to the atlas during adaptation. Valanarasu et al. proposed an on‐the‐fly, single‐sample TTA for medical image segmentation,^29^ in which they trained an autoencoder in a self‐supervised manner as the domain prior generator (DPG). The DPG was used to generate a domain code that directly adapted the network according to the features of the test data. However, the training of the DPG required a variety of medical images consisting of different domains and the model is closer to a domain generalization method. In summary, the single‐sample TTA approach, which uses only a single image from the target domain for model updates without the “ground‐truth” label, is more clinically viable but technically challenging.^26^, ^28^, ^29^ Due to the uncertainty and unreliability of updating the numerous model parameters using only a single sample in an unsupervised manner, existing single‐sample TTA methods are prone to model collapse or overfitting.

In general, there are two questions to be answered in the design of single‐sample TTA: 1. which part of the model should be adapted? And 2. how to adapt the model more effectively and stably? In this study, we aim to design an effective model with an enhanced loss function to update the most pertinent parameters for single‐sample TTA. We proposed an adaptive wavelet‐VNet (WaVNet) model that adapted the spectral and spatial features of each specific test sample at test time through a hybrid objective function. In our model, we dynamically embedded the extracted wavelet coefficients into a V‐Net backbone^33^ with a wavelet attention module for test‐time adaptation. V‐Net is a fully convolutional neural network specifically designed for 3D image segmentation using volumetric data, which is a special case of U‐Net. As a spectral domain feature extraction method, the wavelet transform^34^, ^35^, ^36^ has proven robustness against variations in image intensity and quality. It can effectively extract features across different imaging modalities and conditions to improve the representation capability of deep learning models. Fujieda et al.^37^ combined CNNs and wavelet transform into one model to enhance the accuracy of texture image classification. Duan et al.^38^ utilized low‐frequency wavelet coefficients to calculate pooling layers for remote sensing image segmentation. Agnes et al.^39^ combined the U‐Net++ architecture with wavelet pooling to capture both high‐ and low‐frequency information in the medical images. These methods enhanced model representations by manually selecting some or all of the wavelet coefficients. In this study, we incorporated the wavelet transform into our TTA model to suppress noise and preserve the structure of the learned features for domain‐robust segmentation. Specifically, we designed a wavelet attention module, which can be adapted during the test time, to automatically adjust the weights of different wavelet coefficients based on the input to achieve the best segmentation accuracy. Additionally, we observed that adapting a model to various unseen test samples using an unsupervised loss function showed unstable performance: some samples yielded better segmentations after TTA while others did not. This is because unsupervised learning primarily focuses on extracting commonalities from a large dataset, whereas adapting the model to a single sample aims to achieve individualized results. To mitigate this challenge, we integrated three unsupervised objective functions from different perspectives to enhance the effectiveness and robustness of single‐sample TTA. Given that target regions (organs) in medical image segmentation are often confined to a particular region with similar proportions/sizes, we introduced a symmetric shape‐aware loss to regularize the foreground region, which can improve regional consistency in segmentation and avoid model collapse into trivial solutions. Additionally, we developed a Refine model that calculates a structure prior‐based Refine loss, functioning as a segmentation correction tool. It was designed based on the observation that incorrect segmentations on unseen target domains often resemble those from inadequately trained source‐domain models.^28^ Furthermore, we employed an entropy loss to maintain the overall confidence of the segmentation results. As the shape‐aware loss focuses on local regions, the entropy loss promotes global consistency, and the Refine loss further corrects the segmentations through learned shape priors, these multi‐objective loss terms can complement and enhance each other. Using these three unsupervised loss terms as guidance, we updated the parameters of the wavelet attention module (for spectral feature adaption) and batch normalization (BN) layers (for spatial feature adaptation) in the adaptive WaVNet during single‐sample TTA. We evaluated the proposed method on multidomain, multisite liver and prostate MRI datasets for segmentation, and compared their performance against several state‐of‐the‐art methods. Experimental results show that employing the hybrid objective function for the adaptive WaVNet yields consistent and stable performance improvements. The main contributions of this work can be summarized as follows:

1) We integrated spectral wavelet coefficients into spatial feature learning and proposed an adaptive WaVNet for dynamic TTA to a single test sample. This approach is well‐suited for clinical applications where the pretrained model does not perform well due to unknown test‐time domain gaps.
2) We designed a hybrid objective function, including a shape loss that focuses on the local foreground segmentation, an entropy loss that enhances global consistency, and a Refine loss that further corrects the segmentation using learned shape priors, to enhance the effectiveness and stability of single‐sample TTA. We also demonstrated that ensembling the three losses for TTA yielded more stable results.
3) We conducted extensive experiments across different target domains to demonstrate that the adaptive WaVNet can significantly improve the performance of a pretrained model, and outperform state‐of‐the‐art TTA methods.

## 2 MATERIALS AND METHODS

**FIGURE 1:**
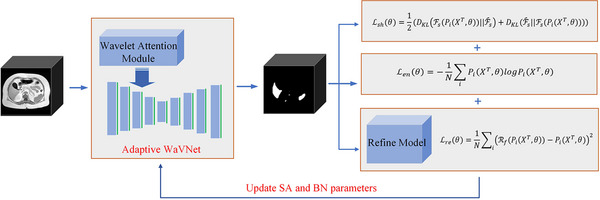
Overview of the adaptive WaVNet framework for TTA. BN, batch normalization layers; TTA, test‐time adaptation; SA, sub‐band attention blocks.

The adaptive WaVNet aims to address domain shifts within the same modality, making it an intramodality adaptation method. Intermodality adaptations (for instance CT to MR) usually involve larger domain gaps that are challenging for test‐time adaptation, especially for the single‐sample scenarios. Thus, our study focuses on the intramodality adaptations, including adaptations between MR scans of different sequences, or MR scans performed across different medical centers/sites. The overall framework is shown in Figure 1, which comprises the adaptive WaVNet model and the hybrid objective function. The corresponding details are introduced in the following, along with the algorithm description of the proposed TTA method.

### 2.1 The adaptive WaVNet

As a geometrical analysis method, the wavelet transform uses different filters to extract spectral features of images across various scales. A multilevel wavelet transform can be expressed as follows^37^:

(1) $$F_{L,n+1}=\left(F_{L,n}*k_{L,n}\right)↓2,$$

(2) $$F_{H,n+1}=\left(F_{L,n}*k_{H,n}\right)↓2,$$

Where *k_L,n_* and *k_H,n_* represent the low‐pass and high‐pass filters, with *n* denoting the wavelet level. $F_{L,n}$, $F_{L,n+1}$, and $F_{H,n+1}$ denote the calculated low‐frequency and high‐frequency coefficients/sub‐bands (*F_L,0_* denotes the input 3D image, *X*).

**FIGURE 2:**
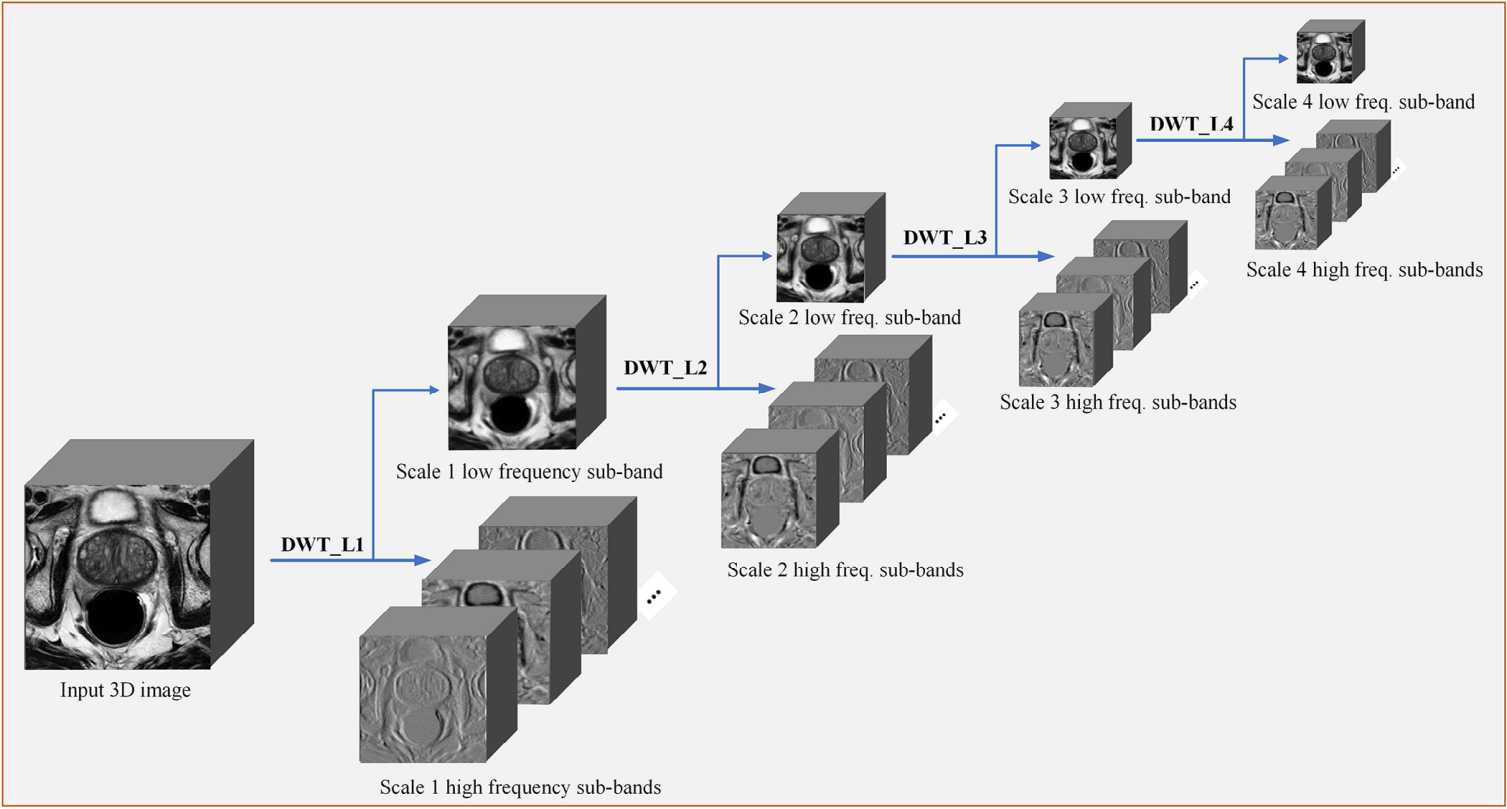
Illustration of the multilevel wavelet transform on a 3D image. DWT_LX: discrete wavelet transform on level X. DWT, discrete wavelet transform.

Figure 2 illustrates the use of multilevel wavelet transform to calculate wavelet coefficients of different scales from a 3D image. Specifically, given a 3D input image $X\in R^{N\times N\times C}$ and a wavelet decomposition level *n*, applying the discrete wavelet transform (DWT) on the image generates the scale‐1 low‐frequency and high‐frequency sub‐bands. Based on the extracted scale‐1 low‐frequency sub‐band, further applying DWT yields scale‐2 low‐frequency and high‐frequency sub‐bands, and the low‐frequency sub‐bands are similarly passed along for information extraction by the following higher scales. Based on the scheme, four levels/scales of low‐frequency and high‐frequency sub‐bands can be obtained as {$F_{L,1},F_{H,1}\},\;\left\{F_{L,2},F_{H,2}\right\},\left\{F_{L,3},F_{H,3}\right\},\;\text{and}\;\{F_{L,4},F_{H,4}$}. These sub‐bands provide a richer representation of the original image from the frequency space, which can augment and supplement the features extracted by traditional spatial filters used in conventional convolutional neural networks.

On the other hand, a typical V‐Net comprises multiple convolutional, down‐sampling, and up‐sampling layers. The convolution and down‐sampling can be represented mathematically as follows:

(3) $$F_{l+1}=\left(F_{l}*K\right)↓p,$$

where *K* denotes the convolutional filters, the symbol ↓ represents the down‐sampling operator, and *p* denotes the stride of down‐sampling. When *p *< 1, the down‐sampling turns into up‐sampling. The symbol *l* denotes the network layer, and *F*_0_ equals the input 3D image *X*.

From the perspective of signal filtering, the wavelet transform and convolutional neural networks, such as V‐Net, have unified mathematical mechanisms,^40^, ^41^, ^42^ as shown in Equations ((1), (2), (3)). The wavelet transform and V‐Net perform similar filtering operations on images, but from the spectral and spatial domain perspectives, respectively. Existing studies^37^, ^43^, ^44^ have successfully incorporated wavelet transform into deep neural networks to boost model feature learning. Most of these studies involved the manual selection of some or all wavelet coefficients for feature augmentation. Considering that low‐frequency coefficients offer a smooth representation of images capturing general intensity/shapes, while high‐frequency sub‐bands encompass more detailed structures, textures, and also some noise,^38^, ^45^ it is hard to manually determine and generalize which sub‐bands are most suitable for feature learning and TTA. Therefore, in this study we introduced a channel attention mechanism, denoted as the wavelet attention module, to allow the model to autonomously select the optimal spectral sub‐bands during pretraining and TTA.

**FIGURE 3:**
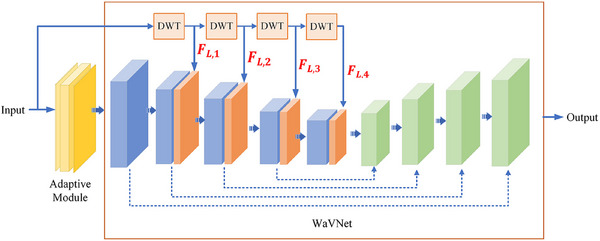
Details of the adaptive WaVNet. DWT_LX: discrete wavelet transforms on level X. During the test time, the parameters of SA and BN layers are adapted with the remaining parameters frozen. BN, batch normalization layers; DWT, discrete wavelet transform; SA, sub‐band attention blocks.

The wavelet attention module is composed of four level DWTs and four sub‐band attention blocks (SA). SA is composed of a pooling layer and two fully connected layers to enable channel attention.^46^ The low‐ and high‐frequency wavelet sub‐bands at each scale were extracted by the DWTs and then automatically weighted by the SAs. The weighted wavelet features from the four scales were concatenated into the 2nd, 3rd, 4th, and 5th layers of the V‐Net encoder to combine with the spatial features and generate the adaptive WaVNet (Figure 3). This adaptive and hierarchical spectral feature learning scheme, combined with the spatial learning from V‐Net, enhances the model's representation on a single sample. By adapting the parameters in the wavelet attention module (SA) and in the V‐Net batch normalization layers during the test time, the framework allows the feature learning to dynamically adapt to different target data, thereby improving the representation ability and transferability of the model.

### 2.2 Hybrid objective function

After constructing the adaptive WaVNet, we first trained it on the source domain $\left\{X^{S},Y^{S}\right\}$ and then adapted the model to the target domains $\left\{X^{T}\right\}$ using the hybrid objective function. We integrated three unsupervised loss functions: an entropy loss, a shape‐aware loss, and a Refine loss to address diverse unseen samples. The first entropy loss term in the unsupervised objective function encouraged high global confidence in the segmentation predictions of the target. This was achieved by minimizing the Shannon entropy of the softmax predictions:

(4) $$L_{\mathrm{en}}\left(\theta \right)=-\frac{1}{N}\sum_{i}P_{i}\left(X^{T},\theta \right)\log P_{i}\left(X^{T},\theta \right),$$

where i indicates the voxel index, $X^{T}$ denotes the input 3D target‐domain image, $P_{i}\left(\cdot \right)$ is the segmentation prediction, *N* denotes the number of voxels in a sample, and θ represents the model parameters to be optimized during the test time (including the wavelet sub‐band attention blocks and the V‐Net batch normalization layers).

Existing studies have found that minimizing the entropy loss alone may result in trivial solutions, where the predictions are biased towards the background or the whole body rather than the target organ in a medical image. To address this issue, we designed a symmetrical shape‐aware loss to focus on the foreground region. The class‐ratios prior (the proportion of a region/class in an image) is a simple yet effective shape descriptor,^24^ and was widely used in the field of medical image analysis.^47^ Our symmetric shape‐aware loss is used to adapt the foreground region of the predictions $F_{s}$ to be close to the class‐ratios prior $\hat{F_{s}}$, as follows:

(5) $$\begin{array}{ccc} L_{\mathrm{sh}}\left(\theta \right) & = & \frac{1}{2}(D_{\mathrm{KL}}\left(F_{s}\left(P_{i}\left(X^{T},\theta \right)\right)\left|\right|\hat{F_{s}}\right)\\ & & +D_{\mathrm{KL}}\left(\hat{F_{s}}\left|\right|F_{s}\left(P_{i}\left(X^{T},\theta \right)\right))\right), \end{array}$$

where $F_{s}\left(\cdot \right)$ denote the statistics of foreground class ratios, $\hat{F_{s}}$ is the mean class ratio of the source domain, and $D_{\mathrm{KL}}\left(\cdot \right)$ represents the Kullback–Leibler (KL) divergence.

The class‐ratio $\hat{F_{s}}$ provides weak prior knowledge to guide the segmentation. To further stabilize the TTA, we developed a Refine model to capture additional domain‐invariant shape priors that allow test‐time segmentation correction and model update. The Refine model was motivated by the observation that when source domain models were tested on target domain samples with distribution shifts, the segmentation results often showed poor regional consistency, inaccurate edges, misclassifications, and noise, similar to those obtained from inadequately trained source models.^48^, ^49^ Thus, we used undertrained source models to generate incomplete and noisy segmentation data and combined them with the known “ground truth” to train the Refine model on source domain, which learned to evaluate/fix incorrect segmentations. Specifically, we used different early stopping epochs to derive a variety of source models and used the resulting models to generate incomplete and noisy segmentation results. The Refine model was then trained using the incorrect segmentations as inputs with their corresponding “ground‐truth” labels as predictions. During the training of the Refine model, it gradually learned how to repair incomplete segmentations to match the “ground‐truth” labels. We instantiated the Refine model as a reconstruction network, utilizing encoding and decoding structures to reconstruct and repair the segmentation results.^28^, ^50^, ^51^ During TTA, we used the Refine model to construct a Refine loss $L_{\mathrm{re}}$, which is a mean squared error (MSE) loss between the segmentation predictions $P_{i}\left(\cdot \right)$ and the Refine model‐reconstructed segmentations $R_{f}\left(\cdot \right)$:

(6) $$L_{\mathrm{re}}\left(\theta \right)=\frac{1}{N}\sum_{i}{\left(R_{f}\left(P_{i}\left(X^{T},\theta \right)\right)-P_{i}\left(X^{T},\theta \right)\right)}^{2},$$

where$\;R_{f}\left(\cdot \right)$ denotes the Refine model.

The final objective function $L_{\mathrm{total}}$ for single sample TTA is defined as follows:

(7) $$L_{\mathrm{total}}\left(\theta \right)=\lambda_{1}L_{\mathrm{en}}\left(\theta \right)+\lambda_{2}L_{\mathrm{sh}}\left(\theta \right)+L_{\mathrm{re}}\left(\theta \right),$$

where $\lambda_{1}$ and $\lambda_{2\;}$ denote hyperparameters to balance the loss terms, which were empirically determined to be 0.5 and 1 in this study.

### 2.3 Algorithm implementation

Different from general image segmentation methods, the TTA framework comprised two phases: Training in the source domain and adaptation in the target domain. During the training phase, the adaptive WaVNet and the Refine model were trained using supervised losses within the source domain. In the adaptation phase, the pretrained parameters were inherited and remained fixed except for those in the wavelet attention blocks and BN layers. These parameters were further updated using the hybrid objective function to adapt to each unseen target sample. The complete algorithm is outlined in Algorithm 1.

Algorithm 1 **Adaptive WaVNet for TTA in 3D medical image segmentation.**

**Table:** 

**Input**: Source domain images $X^{S}=\left\{x_{n}^{S}\right|n=1,2,\text{…}N_{S}\}$ and their labels $Y^{S}=\left\{y_{n}^{S}\right|n=1,2,\text{…}N_{S}\}$; target domain images $X^{T}=\left\{x_{m}^{T}\right|m=1,2,\text{…}N_{T}\}$;
**Output**: Segmentation results of target domain images $Y^{T}=\left\{y_{m}^{T}\right|m=1,2,\text{…}N_{T}\}$.
***Training in the source domain***:
***Step 1***: Training adaptive WaVNet: Training the adaptive WaVNet in the source domain (with images and labels) using the Dice similarity loss until convergence.
***Step 2***: Generating training data for the Refine model: Training a variety of adaptive WaVNet models by early stopping with different epochs to generate undertrained segmentation results.
***Step 3***: Training Refine model: Training the Refine model using the undertrained segmentation results and their corresponding “ground‐truth” labels until convergence.
***Adaptation in the target domain***:
***Step 4***: Single‐sample TTA: Freezing all model parameters of adaptive WaVNet except for those within the wavelet attention blocks and V‐Net BN layers. Feeding a test sample into adaptive WaVNet and using the hybrid objective function according to Equation 7 to update the parameters for a predefined epoch number.
***Step 5***: Inference: Obtaining the segmentation result $y_{m}^{T}$ by the optimized adaptive WaVNet.

## 3 EXPERIMENTS

We evaluated the adaptive WaVNet on two 3D medical image segmentation tasks and compared its performance with state‐of‐the‐art TTA methods. Additionally, we conducted an ablation study to investigate the individual contributions of the hybrid objective function for the adaptive WaVNet framework.

### 3.1 Datasets

The liver segmentation dataset: We used the CHAOS liver segmentation dataset^52^ to evaluate Adaptive WaVNet. The CHAOS dataset includes two subsets of different MR sequences: T1 DUAL (40 images) and T2 SPIR (20 images). The T1 DUAL images are further divided into the in‐phase (IP: 20 images) and out‐phase (OP: 20 images) sets, which have the same repetition times but different gradient echo times and can be treated as different domains. The three‐domain data were arranged into six comparison groups (IP (source) to OP (target), OP to IP, IP to T2, OP to T2, T2 to IP, and T2 to OP). For source domain model training, we used 15 randomly selected images from the source domain data for training and the remaining 5 for validation. All images were uniformly resampled to (1.5, 1.5, 1.5) mm^3^, cropped to (256, 256, 64), and normalized to [0, 1].

The prostate segmentation dataset: We used a multisite dataset^11^, ^53^ for prostate segmentation to further validate the proposed method. The dataset includes T2‐weighted MR images collated from seven different clinical sites. We used one dataset (MSD, 22 images for training and 7 for validation) as the source domain, while the other six, unseen clinical sites (A–F: 30/30/19/13/12/12 images) were used as the target domains. For each sample, we resampled the volume's spacing to (0.7, 0.7, 0.7) mm^3^, cropped the image size to (256, 256, 64), and normalized the intensity to [0, 1].

### 3.2 Implementation details

We used the baseline V‐Net as the backbone and applied a four‐level DWT with the daubechies wavelet^54^ to construct the WaVNet, and the adaptive SA blocks in the wavelet attention module were composed of a pooling layer and two levels of fully connected layers. The adaptive WaVNet was trained for 2000 epochs as the pretrained source model, with all trainable parameters unfrozen. We also trained six additional WaVNet models for (30, 40, 50, 60, 70, 80) epochs to generate six undertrained models, and used them to output incomplete segmentations as the training samples of the Refine model. The Refine model was implemented as a four‐layer V‐Net with a sigmoid activation function at the end. The Refine model includes an encoder and a decoder, which can be used to reconstruct and repair the inputs. It was trained for 1000 epochs. For TTA, we froze all parameters except for the wavelet attention blocks and BN layers, and updated these parameters for 200 iterations for each test sample. All training was based on a batch size of 1, with the SGD optimizer and a learning rate of 0.0005. All models were implemented using the PyTorch library version 2.2.1. The experiments were conducted on an NVIDIA GeForce RTX4090 GPU, equipped with 24 GB of memory.

We compared the adaptive WaVNet with ten methods to evaluate the efficacy of our method: (1) TL: We trained a V‐Net model using the source domain's training samples for 2000 epochs, and then fine‐tuned the model on the target domain with five labeled samples. (2) DA: We used the source domain images and labels and 80% of the target domain images to train the model with an unsupervised domain adaptation method,^55^ then tested it on the remaining 20% of the target domain images. (3) V‐Net^33^: We trained a V‐Net model using the source domain's training samples for 2000 epochs, and tested on the target domains directly. (4) WaVNet: We trained a WaVNet using the source domain data and tested on the target domains without test‐time fine‐tuning. (5) TENT^30^: After training a V‐Net model on the source domain, we performed TTA on the target domain (based on the whole test set) using the entropy minimization loss for 10 epochs. (6) regional nuclear‐norm and contour regularization (RNCR)^27^: We used regional nuclear norm and contour regularization losses for TTA (based on the whole test set). (7) sharpness‐aware and reliable (SAR)^31^ entropy minimization: We used a sharpness‐aware and reliable entropy minimization method to optimize the BN layers for model adaptation during the testing phase (based on the whole test set). (8) DAE ^28^: We used the reported noisy segmentation generation methods to train a denoising autoencoder (DAE), and used it to adapt an image normalization network for single‐sample TTA. (9) TTAS^25^: We used the shape‐moment‐based loss for single‐sample TTA. (10) Refine V‐Net: We used the Refine loss to adapt the V‐Net for single‐sample TTA (no wavelet incorporation). We performed the nonparametric Wilcoxon signed‐rank test between our method (Adaptive WaVNet) and all the other methods (except for the TL and DA methods due to testing sample size differences), and applied the Bonferroni correction to correct for multiple comparisons.

### 3.3 Ablation study

To evaluate the effectiveness of the three loss terms within the hybrid objective function, we performed an ablation study using six different combinations of losses for single‐sample TTA on the prostate dataset. We compared the entropy loss $L_{\mathrm{en}}\left(\theta \right)$, the shape‐aware loss $L_{\mathrm{sh}}\left(\theta \right)$, the Refine loss $L_{\mathrm{re}}\left(\theta \right)$, the entropy loss combined with the shape‐aware loss $L_{\mathrm{en}}\left(\theta \right)+L_{\mathrm{sh}}\left(\theta \right)$, the shape‐aware loss combined with the Refine loss $L_{\mathrm{sh}}\left(\theta \right)+L_{\mathrm{re}}\left(\theta \right)$, and the total loss $L_{\mathrm{total}}\left(\theta \right)$, separately.

## 4 RESULTS

### 4.1 Illustration of wavelet sub‐band features and the optimized TTA weightings

**FIGURE 4:**
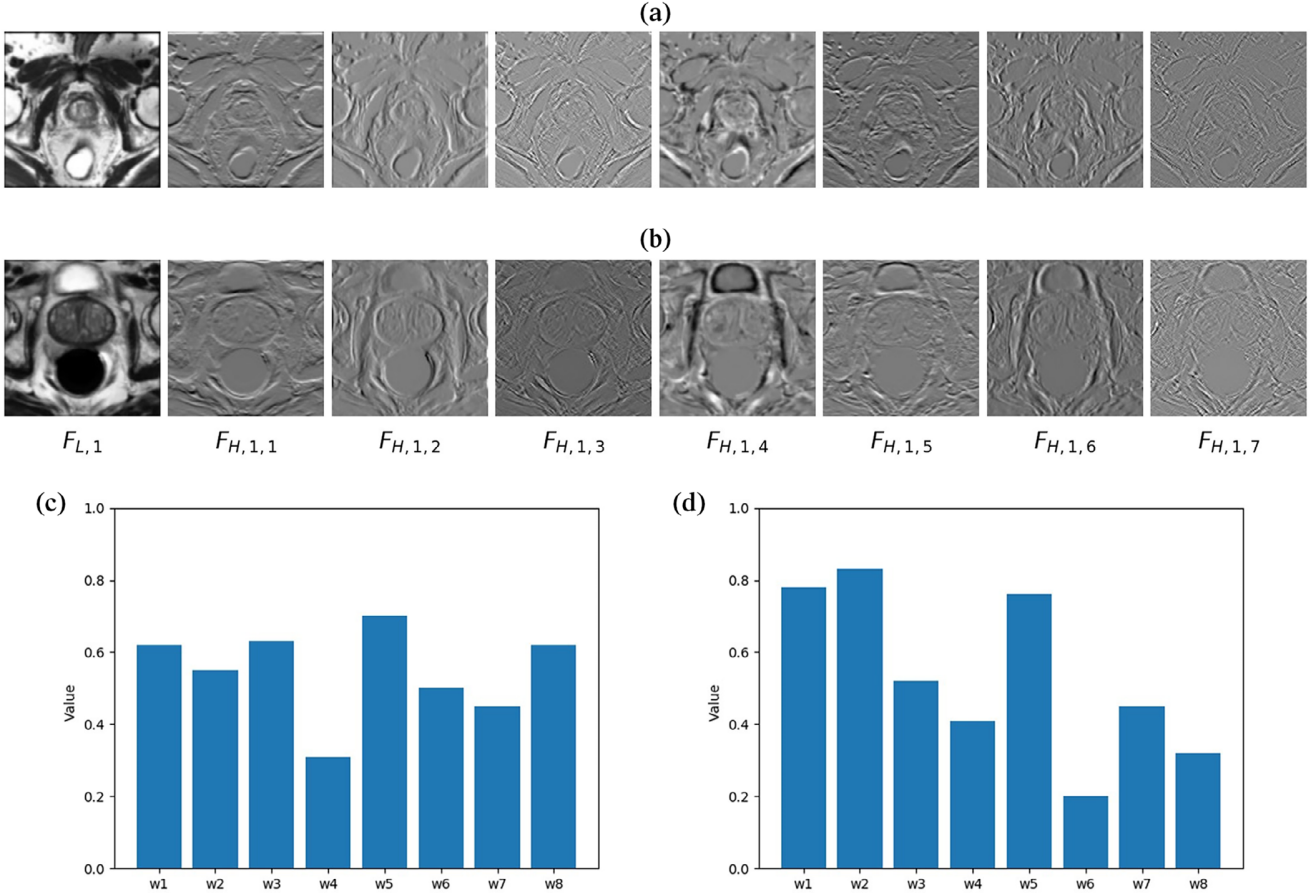
The eight level‐1 wavelet sub‐bands of two sample slices from the prostate dataset (a and b) and their attention weights (c and d, respectively) optimized after the TTA. TTA, test‐time adaptation.

To evaluate the effectiveness of the wavelet attention module, we displayed the eight level‐1 wavelet sub‐bands and the corresponding attention weights after TTA on two prostate slice samples, as shown in Figure 4. The results indicate that the sub‐bands of the wavelet model can capture different intensity and structural information, and the adaptive WaVNet model automatically learned different attention weights for low‐ and high‐frequency sub‐bands during TTA, which is difficult to determine manually.

### 4.2 Liver segmentation results

The mean (± SD) DSC and HD95 results for liver segmentation, obtained using eleven different methods across three target domain datasets, are detailed in Table 1. From the experimental results, it can be seen that fine‐tuning the model using target domain data with labels (TL) outperformed the unsupervised domain adaptation algorithms. When including a large amount of target domain data (80% of all target domain cases) in training DA, the DA model was able to capture the domain gaps to yield better performance than that of single‐sample test‐time adaptation. However, in actual clinical scenarios, the access to labeled target domain data or a large set of target domain images can be prohibited or unrealistic, and the actual domain gap may not be known until the test time. Correspondingly, the TTA methods offer more realistic and convenient alternatives to the TL/DA methods, although with sacrificed accuracy. Serving as the no‐adaptation benchmark, V‐Net in Table 1 yielded the poorest results among all, showing the impacts of domain shifts. Using the wavelet‐incorporated V‐Net, WaVNet offered better segmentation results than V‐Net with wavelet‐guided feature learning, while still suffering from domain shifts and the lack of TTA. The seven TTA methods all outperformed V‐Net, demonstrating the benefits of adapting the pre‐trained model to unseen target data. Compared to TENT, RNCR increased DSC by 2.56% (reported in absolute terms, the same throughout the study) due to the introduction of two new loss functions. SAR enhanced the entropy loss with the sharpness‐aware loss, resulting in a slight improvement in segmentation results than TENT. The DSC of DAE was 7.39% higher than that of V‐Net, benefiting from the incorporation of extra segmentation denoising modules. TTAS adapted the pretrained model with a shape‐moment‐based loss, achieving more accurate segmentations than DAE. The mean DSC of Refine V‐Net was higher than TTAS with the help of Refine loss. However, compared to the existing state‐of‐the‐art TTA methods, our adaptive WaVNet method achieved the highest segmentation accuracy. Compared with TENT/RNCR/SAR, which were adapted collectively using the whole test dataset, adaptive WaVNet was only adapted individually using each test sample, a significantly more challenging task. However, by adapting to sample‐specific traits with the wavelet attention module and the hybrid objective function, it generated substantially better segmentation results. Compared to the DSC coefficient, the HD95 metric in Table 1 is more sensitive to contour edge inaccuracy. The proposed adaptive WaVNet method showed much better results compared with the existing test‐time domain adaptation methods for HD95. For both DSC and HD95, Adaptive WaVNet showed statistically significant differences compared with the eight other methods, based on the Wilcoxon signed‐rank tests with Bonferroni multiplicity correction. The *p‐*values were smaller than 10^−3^, showing that the adaptive wavelet‐VNet method provided better segmentation accuracy than the other methods except for TL and DA.

**TABLE 1:** Average ± SD DSC (%) and HD95 (mm) of eleven different methods on the liver dataset.

		Adaptation between different source and target domains for the liver dataset							
Methods	Metrics	IP to OP	OP to IP	IP to T2	OP to T2	T2 to IP	T2 to OP	Mean	*p*‐Value
TL	DSC (%) ↑ HD95 (mm) ↓	92.22 ± 0.54 2.55 ± 1.64	93.05 ± 1.23 1.43 ± 1.27	90.68 ± 2.04 2.08 ± 1.45	90.79 ± 1.85 2.20 ± 1.01	91.23 ± 1.29 1.86 ± 1.04	92.33 ± 1.08 1.79 ± 1.06	91.72 ± 1.89 1.99 ± 1.43	– –
DA	DSC (%) ↑ HD95 (mm) ↓	90.01 ± 1.33 2.27 ± 1.88	90.23 ± 1.74 3.23 ± 0.97	89.88 ± 3.25 2.94 ± 1.53	90.52 ± 2.10 2.81 ± 1.20	88.37 ± 1.54 1.75 ± 1.34	91.52 ± 0.97 2.62 ± 1.05	90.08 ± 2.13 2.60 ± 1.57	– –
V‐Net	DSC (%) ↑ HD95 (mm) ↓	79.77 ± 2.72 26.33 ± 6.29	65.33 ± 4.55 56.11 ± 5.68	61.78 ± 9.57 52.31 ± 5.54	62.46 ± 6.40 68.35 ± 6.53	65.12 ± 7.73 59.93 ± 9.62	63.57 ± 5.68 45.42 ± 5.40	66.34 ± 8.71 51.41 ± 6.01	*p *< 10^−4^ *p *< 10^−3^
WaVNet	DSC (%) ↑ HD95 (mm) ↓	85.58 ± 6.48 13.57 ± 6.32	71.33 ± 7.03 25.69 ± 5.17	66.12 ± 6.35 25.56 ± 7.08	67.49 ± 8.05 19.77 ± 9.30	69.04 ± 7.38 23.78 ± 8.55	64.39 ± 8.73 25.15 ± 9.41	70.65 ± 7.05 22.25 ± 7.58	*p *< 10^−4^ *p *< 10^−3^
TENT	DSC (%) ↑ HD95 (mm) ↓	84.90 ± 3.33 24.98 ± 6.38	76.61 ± 3.62 49.39 ± 7.44	60.91 ± 6.29 78.58 ± 4.51	64.87 ± 4.70 62.95 ± 6.58	67.51 ± 5.65 38.25 ± 8.89	64.10 ± 6.44 37.41 ± 6.57	69.81 ± 8.68 48.59 ± 7.72	*p *< 10^−4^ *p *< 10^−3^
RNCR	DSC (%) ↑ HD95 (mm) ↓	84.49 ± 2.42 24.06 ± 5.72	77.94 ± 3.49 33.25 ± 4.30	64.45 ± 5.54 63.41 ± 6.63	69.54 ± 5.25 42.91 ± 8.57	71.89 ± 3.34 35.58 ± 5.41	68.93 ± 4.27 37.55 ± 4.86	72.37 ± 6.21 39.46 ± 5.82	*p *< 10^−4^ *p *< 10^−3^
SAR	DSC (%) ↑ HD95 (mm) ↓	85.01 ± 6.78 11.09 ± 4.26	73.57 ± 8.03 19.49 ± 6.27	64.19 ± 8.32 55.15 ± 10.82	67.44 ± 9.02 25.03 ± 5.98	69.93 ± 7.18 22.54 ± 6.53	65.33 ± 8.22 26.25 ± 7.08	70.91 ± 7.55 26.59 ± 5.84	*p *< 10^−4^ *p *< 10^−3^
DAE	DSC (%) ↑ HD95 (mm) ↓	84.19 ± 3.27 9.53 ± 4.78	75.98 ± 5.02 14.48 ± 6.23	67.05 ± 8.24 54.58 ± 12.32	70.53 ± 7.84 32.19 ± 9.02	75.21 ± 9.37 20.32 ± 7.35	69.44 ± 6.20 32.28 ± 9.08	73.73 ± 6.54 27.23 ± 7.12	*p *< 10^−4^ *p *< 10^−3^
TTAS	DSC (%) ↑ HD95 (mm) ↓	85.98 ± 4.32 11.15 ± 4.06	76.82 ± 5.44 13.39 ± 5.52	69.02 ± 5.39 32.80 ± 9.73	70.05 ± 4.79 21.69 ± 7.64	75.42 ± 5.18 18.14 ± 7.43	70.82 ± 5.54 20.08 ± 7.11	74.68 ± 4.98 19.54 ± 6.29	*p *< 10^−4^ *p *< 10^−3^
Refine V‐Net	DSC (%) ↑ HD95 (mm) ↓	**86.56 ± 1.45** 11.52 ± 5.18	**82.45 ± 2.58** 12.60 ± 5.17	65.50 ± 6.26 32.79 ± 3.50	73.68 ± 4.48 18.37 ± 3.07	75.43 ± 3.53 25.06 ± 6.32	70.32 ± 4.22 28.44 ± 3.46	75.65 ± 7.08 21.46 ± 7.92	*p *< 10^−4^ *p *< 10^−3^
Adaptive WaVNet	DSC (%) ↑ HD95 (mm) ↓	85.52 ± 5.20 **7.52 ± 5.83**	79.15 ± 6.35 **11.03 ± 5.68**	**75.21 ± 6.57** **25.97 ± 6.24**	**78.08 ± 4.79** **16.29 ± 7.32**	**77.38 ± 5.61** **19.02 ± 5.73**	**73.25 ± 6.14** **13.31 ± 7.46**	**78.10 ± 5.23** **15.52 ± 5.84**	**–** **–**

*Note*: The arrows are pointing in the direction of improved accuracy. TL, DA, V‐Net, WaVNet, TENT, RNCR, SAR, DAE, TTAS, and Refine V‐Net are the comparison methods described in Section 3.2. ‘‘XX to YY’’ denotes training on the ‘‘XX’’ dataset, and testing on the ‘‘YY’’ dataset. All training and testing datasets (IP, OP, T2) contain 20 samples, except for TL and DA. For TL, all training used 20 samples from the source domain and 5 samples from the target domain (fine‐tuning), and the testing used 15 samples from the target domain. For DA, all training used 36 samples (20 from the source domain and 16 from the target domain), and all testing used 4 samples from the target domain. Statistical tests based on Wilcoxon signed‐rank tests with Bonferroni correction showed Adaptive WaVNet provides statistically significant improvements over the other methods except for the TL and DA methods (*p *< 10^−3^ for all cases). Statistical testing was not performed for the TL and DA results due to the sample size differences.

Abbreviations: DSC, Dice coefficient; HD95, Hausdorff distance; WaVNet, wavelet‐VNet.

Bold indicates the best results of each group experiments.

**FIGURE 5:**
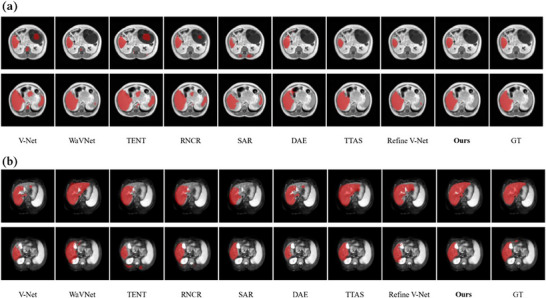
Visual comparison of segmentation results for two different slices using different TTA methods on the liver dataset (*a*: in‐phase T1 to out‐phase T1; *b*: out‐phase T1 to T2.). V‐Net, WaVNet, TENT, RNCR, SAR, DAE, TTAS, and Refine V‐Net are the comparison methods described in Section 3.2. ‘Ours’ indicates the adaptive WaVNet model developed in this study. DAE, denoising autoencoder; GT, ground‐truth reference; TTA, test‐time adaptation; WaVNet, wavelet‐VNet.

The qualitative results of liver segmentation are shown in Figure 5 for two adaptation scenarios (subfigures a and b). Each subfigure presents the segmentations at two different slice locations using various methods. The segmentation maps of V‐Net exhibit considerable noise and discontinuities, while the results of WaVNet are better than V‐Net. With TTA, the results of TENT, RNCR, and SAR show improvements compared to V‐Net, although segmentation islands and discontinuities remain. DAE, TTAS, and Refine V‐Net further reduce noise and over‐segmented areas. Overall, the adaptive WaVNet segmentation results (Ours) are closest to the “ground‐truth” reference, demonstrating the effectiveness of combining adaptive feature learning with multi‐objective function‐guided test‐time domain adaptation.

### 4.3 Prostate segmentation results

**FIGURE 6:**
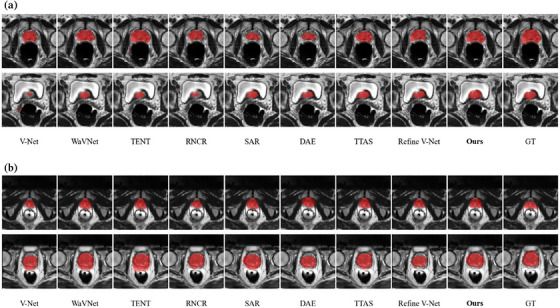
Visual comparison of segmentation results for two different slices using different TTA methods on two prostate datasets (*a*: MSD to Site B; *b*: MSD to Site F.). V‐Net, WaVNet, TENT, RNCR, SAR, DAE, TTAS, and Refine V‐Net are the comparison methods described in Section 3.2. ‘‘Ours’’ indicates the adaptive WaVNet model developed in this study. DAE, denoising autoencoder; GT, ground‐truth reference; TTA, test‐time adaptation; WaVNet, wavelet‐VNet.

The quantitative results, including the mean (± SD) DSC and HD95 on six different target domains for the prostate dataset, are shown in Table 2. Similar to the liver study, the results of TL were better than those of the TTA methods. Although DA used 80% of the target samples, its average DSC was only 1.24% higher than the proposed method using a single sample for TTA. The poor performance of V‐Net indicates significant domain shifts between different sets. Compared with V‐Net, WaVNet improved segmentation accuracy by embedding multiscale wavelet features into the V‐Net. Across all results, models adapted to target domains using different TTA methods consistently outperformed the model without TTA. Supported by the wavelet attention and the hybrid objective function, our method achieved the best segmentation results for single‐sample adaptation. Similarly, Adaptive WaVNet showed statistically significant differences compared with the eight other methods, based on the Wilcoxon signed‐rank tests with Bonferroni multiplicity correction. The *p‐*values were smaller than 10^−3^. Figure 6 displays the segmentation maps at two different slice locations using nine methods on two prostate datasets. Similarly, adaptive WaVNet generated the best segmentation results.

**TABLE 2:** Average ± S.D. DSC (%) and HD95 (mm) of eleven different methods on the prostate dataset.

		Adaptation from a single source domain MSD to multitarget domains (A‐F) on the prostate dataset							
Methods	Metrics	Site A (30)	Site B (30)	Site C (19)	Site D (13)	Site E (12)	Site F (12)	Mean	*p*‐Value
TL	DSC (%) ↑ HD95 (mm) ↓	90.02 ± 1.53 2.24 ± 1.09	81.54 ± 3.79 4.85 ± 3.27	84.08 ± 2.38 5.73 ± 2.62	85.59 ± 2.30 6.84 ± 2.71	83.62 ± 3.88 7.08 ± 3.57	87.51 ± 1.50 4.49 ± 2.12	85.39 ± 2.03 5.21 ± 2.57	*–* *–*
DA	DSC (%) ↑ HD95 (mm) ↓	89.70 ± 3.53 3.97 ± 1.24	75.47 ± 5.78 17.62 ± 5.66	77.65 ± 5.79 13.65 ± 4.21	85.23 ± 2.50 4.25 ± 1.02	72.73 ± 6.88 14.85 ± 3.53	86.80 ± 2.69 5.38 ± 2.42	81.26 ± 3.25 9.95 ± 2.59	*–* *–*
V‐Net	DSC (%) ↑ HD95 (mm) ↓	83.96 ± 3.36 12.11 ± 3.16	53.51 ± 6.55 37.76 ± 8.24	54.79 ± 4.29 54.05 ± 9.89	76.21 ± 6.37 51.25 ± 6.51	55.63 ± 5.52 62.88 ± 7.41	64.41 ± 6.35 19.05 ± 5.52	64.75 ± 5.86 39.51 ± 6.33	*p *< 10^−4^ *p *< 10^−3^
WaVNet	DSC (%) ↑ HD95 (mm) ↓	86.52 ± 3.24 11.05 ± 4.08	64.12 ± 5.79 28.83 ± 3.17	69.03 ± 5.03 43.25 ± 7.82	78.22 ± 7.42 46.07 ± 5.62	69.52 ± 7.08 45.07 ± 4.98	73.07 ± 6.73 17.13 ± 4.27	73.41 ± 5.63 29.07 ± 4.51	*p *< 10^−4^ *p *< 10^−3^
TENT	DSC (%) ↑ HD95 (mm) ↓	82.56 ± 4.23 13.52 ± 5.23	62.19 ± 6.42 24.07 ± 6.57	56.98 ± 4.31 40.82 ± 6.36	76.05 ± 5.55 38.59 ± 7.42	58.79 ± 6.79 43.79 ± 9.35	68.21 ± 8.27 13.88 ± 6.33	67.46 ± 6.22 29.11 ± 7.07	*p *< 10^−4^ *p *< 10^−3^
RNCR	DSC (%) ↑ HD95 (mm) ↓	83.78 ± 3.51 11.22 ± 4.09	63.24 ± 4.18 19.74 ± 5.16	57.09 ± 5.82 30.85 ± 6.52	75.31 ± 6.19 29.15 ± 10.46	59.30 ± 8.38 32.06 ± 5.20	69.82 ± 6.12 15.23 ± 4.38	68.09 ± 5.84 23.04 ± 6.08	*p *< 10^−4^ *p *< 10^−3^
SAR	DSC (%) ↑ HD95 (mm) ↓	83.22 ± 4.72 10.08 ± 3.53	63.89 ± 8.36 16.76 ± 6.29	58.80 ± 5.79 25.26 ± 5.24	77.49 ± 5.25 21.46 ± 5.54	60.93 ± 7.39 32.13 ± 8.43	69.27 ± 5.26 14.06 ± 6.53	68.93 ± 6.12 19.25 ± 6.58	*p *< 10^−4^ *p *< 10^−3^
DAE	DSC (%) ↑ HD95 (mm) ↓	84.85 ± 3.92 12.02 ± 4.37	69.97 ± 7.73 15.54 ± 5.27	67.56 ± 6.09 17.09 ± 7.82	79.01 ± 6.12 19.08 ± 4.45	66.22 ± 9.50 28.83 ± 7.12	76.45 ± 5.23 15.08 ± 4.16	73.34 ± 7.04 17.71 ± 5.13	*p *< 10^−4^ *p *< 10^−3^
TTAS	DSC (%) ↑ HD95 (mm) ↓	**89.23 ± 3.33** 10.64 ± 3.15	70.58 ± 5.18 18.61 ± 5.69	69.62 ± 4.34 19.08 ± 5.42	81.00 ± 5.76 16.73 ± 6.32	67.46 ± 8.38 22.08 ± 5.93	77.89 ± 3.91 14.81 ± 6.25	75.96 ± 5.26 16.19 ± 4.03	*p *< 10^−4^ *p *< 10^−3^
Refine V‐Net	DSC (%) ↑ HD95 (mm) ↓	84.57 ± 3.39 8.532 ± 2.20	63.21 ± 4.21 12.47 ± 5.21	65.27 ± 7.40 14.79 ± 5.09	78.86 ± 5.32 21.89 ± 5.24	65.05 ± 7.54 27.11 ± 6.31	70.45 ± 4.23 13.28 ± 3.39	71.24 ± 7.94 16.35 ± 6.25	*p *< 10^−4^ *p *< 10^−3^
Adaptive WaVNet	DSC (%) ↑ HD95 (mm) ↓	88.91 ± 2.09 **4.11 ± 4.29**	**74.38 ± 7.96** **12.19 ± 4.87**	**76.53 ± 5.56** **11.81 ± 4.330**	**83.25 ± 5.07** **9.72 ± 5.18**	**76.01 ± 6.21** **10.64 ± 4.53**	**81.04 ± 4.23** **6.78 ± 3.04**	**80.02 ± 4.39** **9.18 ± 3.47**	**_–_** **_–_**

*Note*: The arrows are pointing in the direction of improved accuracy. TL, DA, V‐Net, WaVNet, TENT, RNCR, SAR, DAE, TTAS, and Refine V‐Net are the comparison methods described in Section 3.2. The number after each site name showed the test samples included in each dataset, except for the TL and DA methods. For TL, 5 additional samples from the testing dataset were used for source model fine‐tuning, and the remaining was used for testing. For DA, 80% of samples from the testing dataset was combined with the source domain samples for training, and the remaining was used for testing. Statistical tests based on Wilcoxon signed‐rank tests with Bonferroni correction showed Adaptive WaVNet provides statistically significant improvements over the other methods except for the TL and DA methods (*p* < 10^−3^ for all cases). Statistical testing was not performed for the TL and DA results due to the sample size differences.

Abbreviations: DAE, denoising autoencoder; DCS, Dice coefficient; HD95, Hausdorff distance; WaVNet, wavelet‐VNet.

Bold indicates the best results of each group experiments.

### 4.4 Ablation study

**FIGURE 7:**
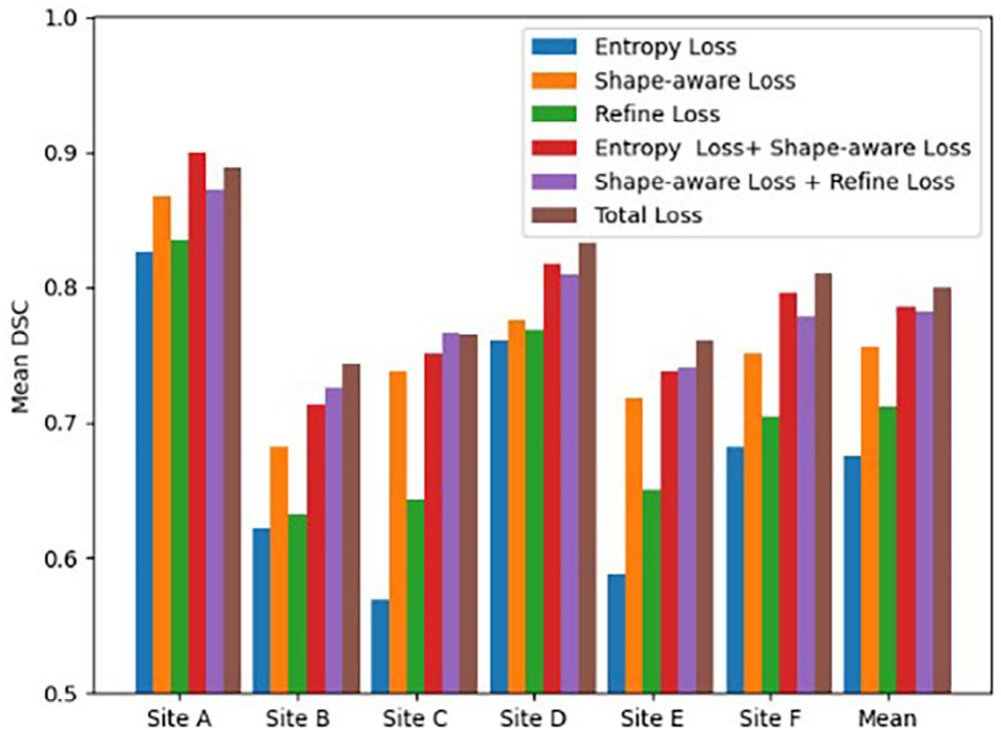
Ablation study of the hybrid objective function on the prostate dataset (Sites A–F), by employing different combinations of the three loss terms. The numbers of test samples included in Sites A–F are 30, 30, 19, 13, 12 and 12, respectively.

**FIGURE 8:**
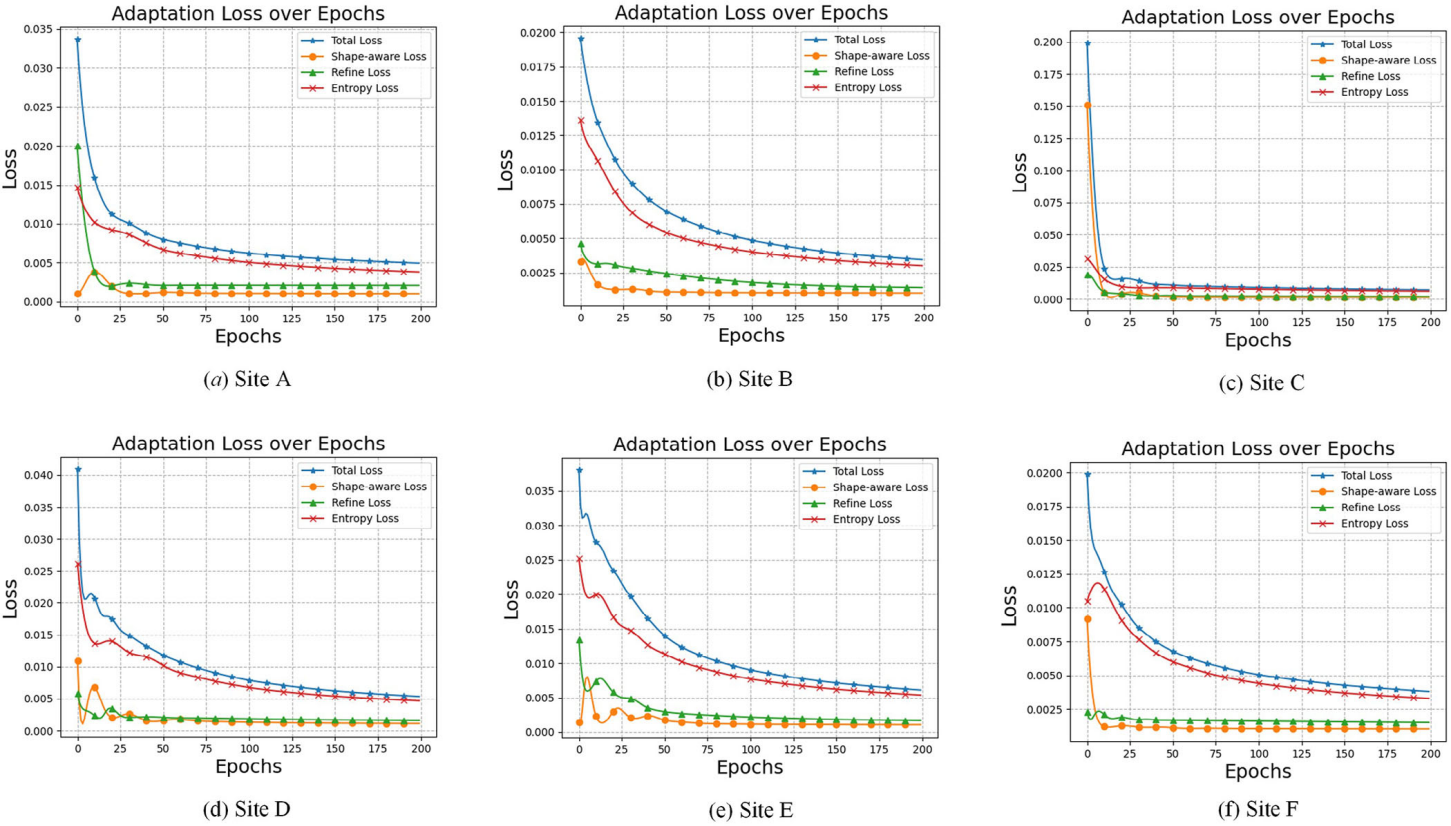
Curves of different loss items on different target domains (prostate datasets Site A∼F) during single‐sample TTA. The curves in each figure correspond to the same TTA process, governed by the total loss, for a single sample from each test site. TTA, test‐time adaptation.

The detailed experimental results for the loss term ablation study are shown in Figure 7. From the experimental results, it can be observed that the combination of the shape‐aware and entropy losses achieved the highest segmentation accuracy on the Site *A* dataset, and the shape‐aware and Refine losses yielded the best results on the Site *C* dataset, and the total loss performed the best across the other site datasets. However, even for Sites *A* and *C*, the total loss provided the second best results that are very close to the first‐ranked results. The plots indicate that the performance of the hybrid objective function is more stable and reliable. To further demonstrate the stability of the total loss, we plotted the change of loss terms during the adaptation process for a single sample randomly drawn from the different test domains, as shown in Figure 8.

From Figure 8 it can be observed that combining the three loss items helps to smooth and stabilize the loss curves, and allows all loss items to converge to a stable state, even if the independent loss items were initialized with different magnitudes and relative rankings in the beginning for different domains.

### 4.5 Time cost analysis

**FIGURE 9:**
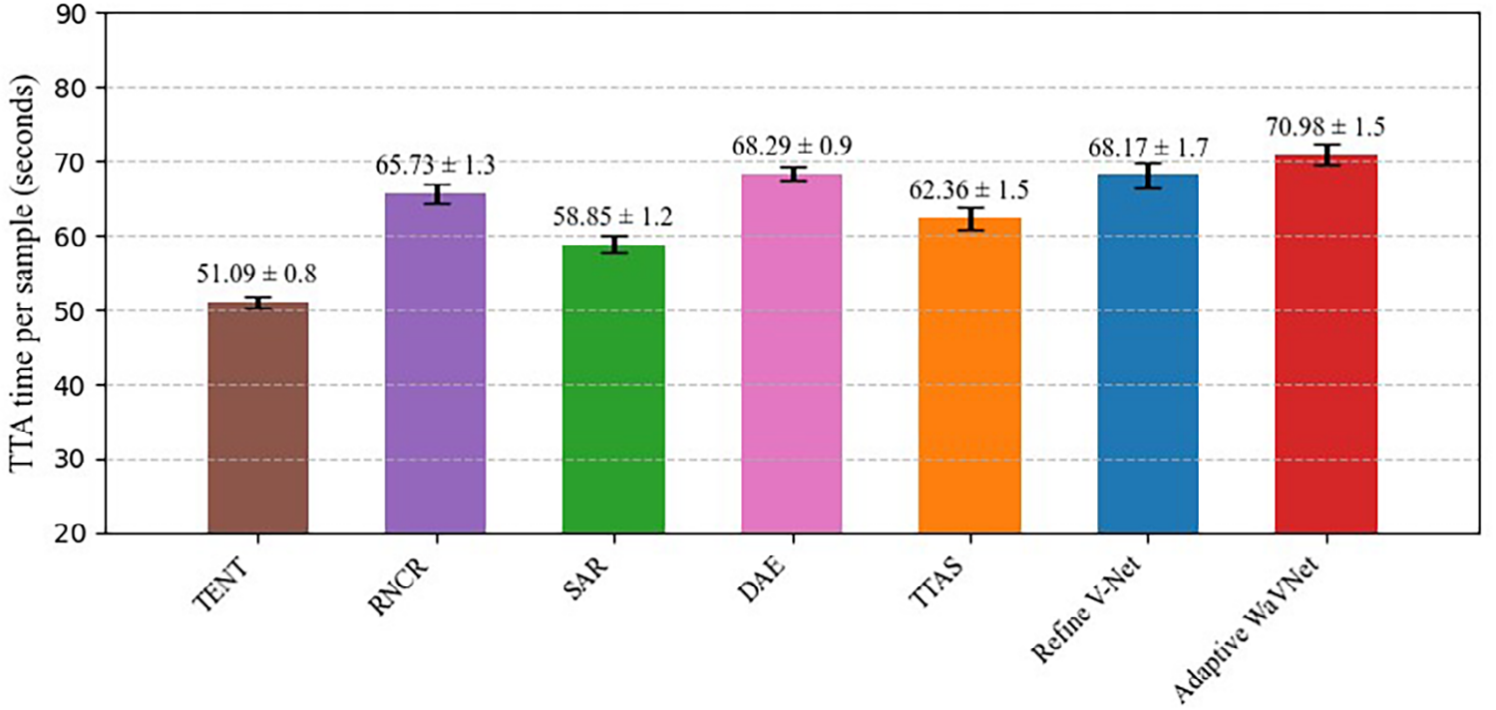
The time cost of different TTA methods, with the mean and standard deviation shown for each method (based on 20 testing samples). TTA, test‐time adaptation.

To compare the time costs of different TTA methods, we performed an analysis on the CHAOS dataset (InPhase to OutPhase adaptation, based on the NVidia RTX4090 GPU). Since the TENT, RNCR, and SAR methods are designed for test‐time adaptation using the full set of testing samples (20 samples), their running time is much longer than the single‐sample TTA methods (DAE, TTAS, Refine V‐Net, and Adaptive WaVNet). For a fair time comparison, we re‐ran the TENT, RNCN, and SAR methods for single‐sample adaptation on each of the 20 testing samples solely for the purpose of time cost comparison, and did not consider the corresponding performance degradations. We calculated the mean (± SD) running time of 20 testing samples for different TTA methods. The results are illustrated in Figure 9.

Among the seven TTA methods, four of them have additional modules (RNCR, DAE, Refine V‐Net, and Adaptive WaVNet). Therefore, their test‐time adaptations took relatively longer time. TENT is the basic TTA method in our experiments, which used only the entropy loss to optimize the BN layers in the VNet, resulting in the lowest adaptation time cost. Our method, Adaptive‐WaVNet, calculated three objective functions and updated the parameters of the wavelet attention module and BN layers, leading to higher time costs. However, the overall time costs among different methods are not substantially different, with a range of 50–70 s.

## 5 DISCUSSION AND CONCLUSION

Most statistical learning algorithms strongly depend on the i.i.d. assumption. However, the test data in real‐world applications can often be out‐of‐distribution (OOD), leading to algorithm performance drops. Moreover, the test data in the target domain are often scarce and unlabeled, rendering supervised model fine‐tuning impractical. To address this challenge, the adaptive WaVNet framework was proposed for single‐sample TTA, which did not require labeled target‐domain data for fine‐tuning and can adapt the model based on a single test‐domain image. Since the model is independently adapted for each test sample, it is not susceptible to the error accumulation and catastrophic forgetting issues encountered by adaptive models continuously updated on accumulated testing samples. By dynamically embedding multiscale wavelet coefficients into the V‐Net encoder, adaptive WaVNet effectively extracted rich features from each unseen sample, thereby enhancing the model's representation. The hybrid objective function allowed WaVNet to be effectively and stably updated during test time, further improving segmentation accuracy. Tested on multidomain liver and prostate datasets, the quantitative and qualitative segmentation results (Table 1, Table 2, Figure 5, and Figure 6) demonstrated that the introduction of wavelet attention enhanced the representation of the baseline V‐Net for singular samples. With wavelet infusion only, the DSC increased from 66.34 ± 8.71% to 70.65 ± 7.05% on the liver dataset and from 64.75 ± 5.86% to 73.41 ± 5.63% on the prostate dataset. After applying TTA with the proposed hybrid objective function, the DSC further increased to 78.10 ± 5.23% on the liver dataset and to 80.02 ± 4.39% on the prostate dataset, which indicated that combining multi‐objective functions could enhance the performance and stability of model adaptation. Furthermore, we compared our method with several TTA methods (RNCR, DAE, and TTAS) specifically designed for medical images and TTA methods (TENT, SAR) from the natural image segmentation field. Among all competitive methods, our proposed method achieved the best segmentation performance across both datasets with multiple target domains and various domain shifts.

Although TL and DA showed some performance improvement over the TTA method (the performance enhancement was less pronounced for the prostate dataset [Table 2]), they require the target domain data and labels (for TL), or the target domain data (for DA) when training the models. The target domain data and the corresponding domain gaps may not be known until the test time, especially in dynamic and changing clinical scenarios. The TL model requires target domain labels, which may be difficult to obtain in real clinical scenarios. Although not requiring labels, the DA model needs to access a set of target domain data to fully gauge the domain gap, which can be difficult to obtain during the training time. In contrast, the TTA‐based method is more convenient to deploy under different clinical scenarios, as the single‐sample TTA enabled unsupervised re‐optimization of the model using only one unlabeled target sample.

The ablation study investigated the impact of various loss terms on model performance. By evaluating singular loss terms and the combinations of different loss terms, we observed that as the number of objective terms increased, the model's segmentation accuracy improved, confirming the intuition that hybrid multi‐objective optimization is more effective than single‐objective optimization in TTA. There could be a most suitable loss term for each individual case, while currently there is no mechanism to selecting them individually and automatically, rendering the multi‐objective approach more practical. For our study, we combined the shape‐aware loss, which focuses on local foreground segmentations; with the entropy loss, which considers global consistency; and the Refine loss, which further refines the segmentation results through learned shape priors, to enhance the effectiveness and stability of single‐sample TTA. Figure 5 shows the loss curves of TTA across six target domains. Although the loss curves exhibited significant differences across different domains, their overall tendency to smooth out during 200 iterations demonstrated a more stable TTA on each sample.

The performance of a single‐sample TTA model is relatively independent of the testing sample size, as the same source model is used to initialize the TTA for each individual testing sample. Its performance, however, can be potentially dependent on the training sample size of the source domain. The exact dependence, however, is difficult to predict due to the potential interplay between the source model and the test time domain gap. Future studies are warranted to evaluate the effect of the source domain training dataset size on the performance of TTA.

Although the adaptive WaVNet model has achieved notable performance improvements, there remain some potential limitations and issues to be addressed. The proposed method in this study was still handling relatively confined domain gaps (across different MR images scanned by different protocols or hardware), and the segmentation results are mostly inferior to those achieved by models trained on labelled data, which calls for further accuracy improvements. For domains with substantially larger distribution shifts, such as those with different modalities (for instance MR images and CT images), the domain gaps cannot be easily suppressed by the mechanisms proposed in this work. Exploring a broader range of multiscale geometric analysis methods could potentially increase the model's robustness against larger domain shifts.^56^ On the other hand, adapting a large model with a single sample in an unsupervised manner can easily lead to issues including training instabilities, over‐fitting risks, and local optimum traps. In this study, we used three objective functions for TTA to mitigate these issues, while there is no guarantee that the model will converge to the global optimum. Additional priors could further help address such issues. In the context of radiotherapy, a patient‐specific prior contouring set almost always exists, which may provide strong prior knowledge to condition and guide the single‐sample TTA.^26^ Additionally, this study focused on single contour segmentation (liver or prostate) for the two datasets. Performing multi‐organ segmentations may introduce additional challenges and opportunities (for instance, additional regularizations can be enforced to reflect prior knowledge regarding the relative locations/distributions of different structures), which remains to be investigated.

In conclusion, by enhancing feature learning with multiscale wavelet coefficients and adaptively adjusting the spatial and spectral features with the hybrid objective function during TTA, adaptive WaVNet demonstrated superior performance over state‐of‐the‐art TTA methods, showing its potential for improving medical image segmentation across varied domains.

## CONFLICT OF INTEREST STATEMENT

The authors declare no conflicts of interest.

## Data Availability

The multidomain liver and prostate datasets used in this study are publicly available for download and use (the liver dataset: https://chaos.grand‐challenge.org/; the prostate dataset: http://medicaldecathlon.com/.).
